# Transcatheter arterial chemoembolization with spherical embolic material for locally advanced breast cancer: first report of HepaSphere™ treatment for primary breast cancer

**DOI:** 10.1259/bjrcr.20150417

**Published:** 2016-11-02

**Authors:** Norifumi Kennoki, Shinichi Hori, Atsushi Hori, Yuki Takeo, Hisashi Oshiro

**Affiliations:** ^1^Department of Radiology, Tokyo Medical University Hachioji Medical Center, Tokyo, Japan; ^2^Department of Radiology, Gate Tower Institute for Image Guided Therapy, Osaka, Japan; ^3^Department of Radiology, Rinku Dejima Clinic, Osaka, Japan; ^4^Department of Pathology, Tokyo Medical University Hospital, Tokyo, Japan

## Abstract

A 57-year-old female was diagnosed as having primary breast cancer (invasive carcinoma of no special type), which was immunohistochemically negative for oestrogen receptor, androgen receptor and human epidermal growth factor receptor Type 2. The main tumour was 54 × 35 mm in size and was located in the internal upper area of the left breast. The tumour had markedly invaded the skin and a daughter nodule was observed in the external upper area of the ipsilateral breast. An enlarged lymph node measuring 12mm in diameter was present in the axilla and an affected parasternal lymph node was also observed. A blood test showed no abnormalities and the patient was negative for tumour markers. We performed three sessions of transcatheter arterial chemoembolization with docetaxel-loaded HepaSphere™. The treatment procedure was successfully performed in all the three sessions. No adverse events higher than Grade 3 were observed. The sizes of the primary lesion and axillary lymph node decreased to 26 × 14 mm (37% reduction) and 10mm, respectively. The parasternal lymph node completely resolved. 2 months later, left total mastectomy and axillary lymph node dissection were performed. The histopathological post-therapy effect was considered to be a mild response (Grade 1a) in the breast lesion and a complete response (Grade 3) in the axillary lymph node. The mean±standard deviation of the minor axis of the vessels embolized with spherical particles was 183.0±96.5 μm. Our results indicate that transcatheter arterial chemoembolization used together with HepaSphere can be an alternative and effective therapy for locally advanced breast cancer.

## Background

The standard treatment for locally advanced breast cancer (LABC) is neoadjuvant systemic chemotherapy together with mastectomy. However, as patients often refuse this treatment for a variety of reasons, the availability of alternative treatments would be desirable. We propose transcatheter arterial chemoembolization (TACE) with spherical embolic material as a treatment choice for primary breast cancer.

## Clinical presentation

A 57-year-old female visited a nearby clinic with a chief complaint of sharp pain and taut skin in the left breast. After examination, the lesion was diagnosed as primary breast cancer (invasive carcinoma of no special type), which was immunohistochemically negative for oestrogen receptor, androgen receptor and human epidermal growth factor receptor Type 2. The cancer clinical stage was T4bN1M0 Stage IIIB (Union for International Cancer Control guidelines). Neoadjuvant systemic chemotherapy together with mastectomy was recommended as a therapeutic option. However, she refused all treatment and left the lesion untreated for approximately 10 months. When the tumour increased in size and the pain became intolerable, she expressed a strong desire to receive minimally invasive treatment, such as TACE. Thus, she was referred to our clinic.

## Imaging findings

**Figure 1.**
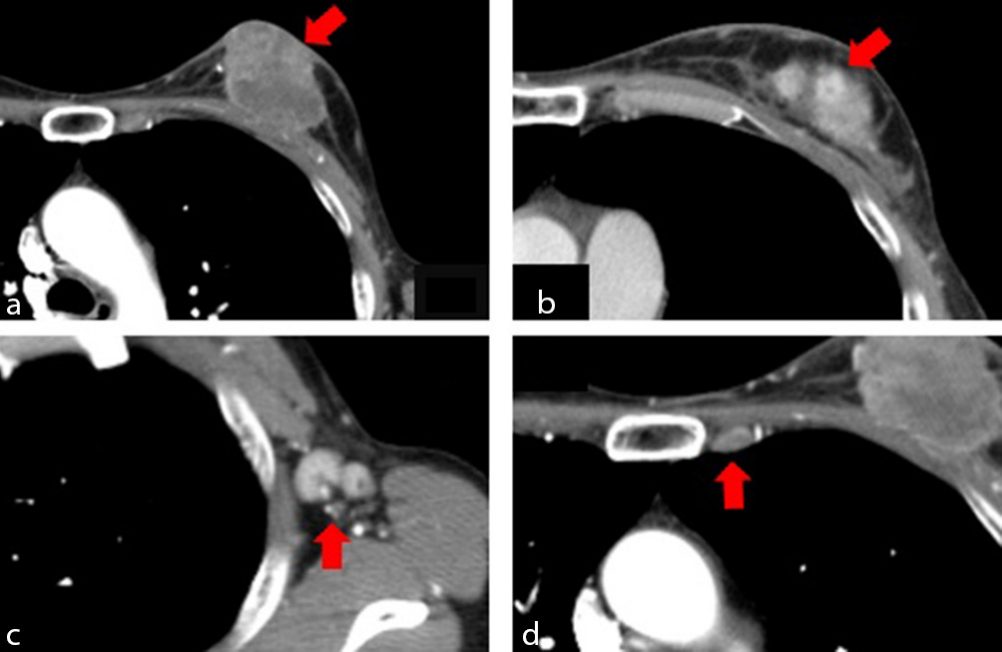
Contrast-enhanced CT images of the patient’s tumour. A 54 mm primary lesion (arrow) is visible on the internal upper area of the left breast, which had invaded the skin (a). A daughter nodule (arrow) visible on the external upper area (b). Enlarged axillary and parasternal lymph nodes (arrows) visible on (c) and (d), respectively.

Contrast-enhanced CT (CECT) was performed and showed a main tumour measuring 54 × 35 mm in size in the internal upper area of the left breast, with apparent skin invasion (Figure 1a). A daughter nodule measuring 8 mm was seen in the external upper area of the ipsilateral breast (Figure 1b). An ipsilateral axillary lymph node measuring 12 mm (Figure 1c), and an affected parasternal lymph node was also observed (Figure 1d). The cancer stage was T4bN3bM0 Stage IIIC.

## Treatment and outcome

TACE was performed once every month for a total of three sessions. The target lesions were the primary breast tumour, daughter lesion, and axillary and parasternal lymph nodes. Treatment was performed using the IVR-CT/Angio system (Toshiba Medical Systems Corporation, Tochigi, Japan), which is an angiography system combined with a CT scanner. Infusion of anticancer drugs followed by embolization with docetaxel-loaded HepaSphere^TM^ was performed in each session. Between treatments, the tumour size and viability were assessed by CECT. Adverse events of the anticancer drug were also routinely assessed. The regimen was changed in the second and third sessions owing to adverse events seen during the previous session (Table 1).

**Table 1.** Treatment regimens, dose of docetaxel-loaded HepaSphere^TM^ used and adverse events observed in our case

Session	Regimen and dose of anticancer drugs	Dose of docetaxel-loaded HepaSphere	Adverse event
1	Cisplatin (30 mg) + docetaxel (20 mg) + fluorouracil (250 mg) + mitomycin C (4 mg)	9.75 mg	Alopecia (Grade 2)
2	Cisplatin (10 mg) + adriamycin (20 mg) + fluorouracil (250 mg) + mitomycin C (4 mg)	7.75 mg	Pigmentation (Grade 1)
3	Cisplatin (10 mg) + adriamycin (10 mg) + fluorouracil (250 mg) + mitomycin C (2 mg)	5.25 mg	None

**Figure 2.**
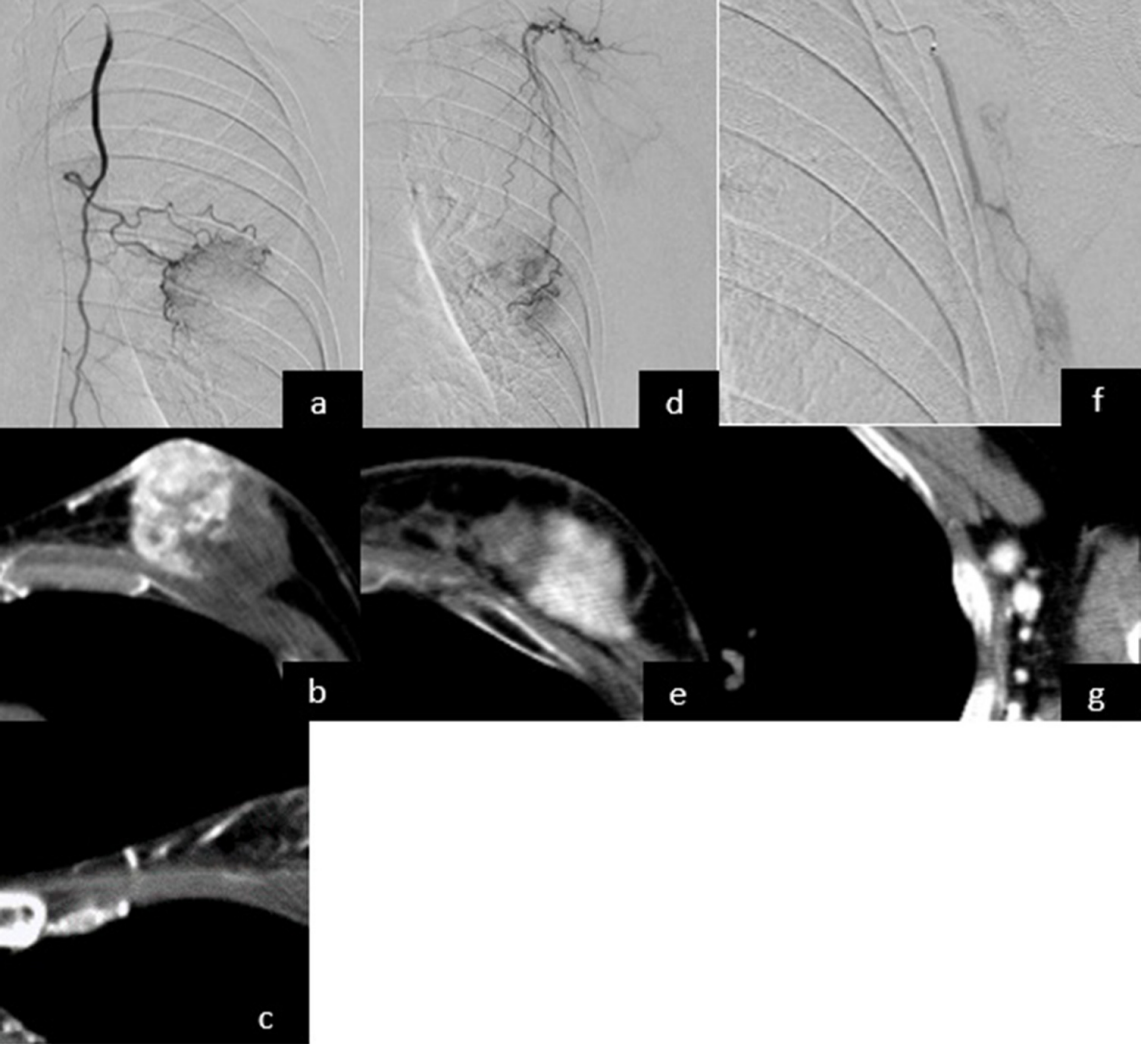
DSA and CTA images taken during the procedure. DSA and CTA images of the left internal mammary artery showing vasculatures of the inner area of the primary lesion (a, b) and parasternal lymph node (c). DSA and CTA images of the left thoracoacromial artery demonstrating staining of the outer part of the primary lesion (d, e). DSA and CTA images of the left thoracodorsal artery confirming the blood supply to the axillary lymph node (f, g). CTA, CT angiography; DSA, digital subtraction angiography.

We catheterized the right femoral artery under local anaesthesia and coaxially inserted a 4-French sheath, a 4-French guiding catheter and a 1.9-French microcatheter. The tip of the guiding catheter was placed to the left of the subclavian artery to gain system stability. The microcatheter was advanced to the arteries feeding the lesions. The left internal thoracic artery fed the inner side of the main tumour and parasternal lymph node (Figure 2a–c). The left thoracoacromial artery fed the outer side of the main tumour (Figure 2d,e), and the left thoracodorsal artery fed the axillary lymph nodes (Figure 2f,g). Before infusion, CT angiography was performed with the IVR-CT/Angio system to confirm whether the catheter was in the correct position to avoid non-target embolization. For each session, one vial of 50–100 µm HepaSphere (25 mg) in the dry state, was diluted with an aqueous solution containing 1 ml of 10% NaCl, 4 ml of contrast material and 10 mg of docetaxel. In the first session, a docetaxel-based regimen by percentage of tumour volume was administered to each feeding artery. The injection rate of the drug was very slow (3 ml min^–1^ for the anticancer drugs and 1 ml min^–1^ for docetaxel-loaded HepaSphere), and the drug was carefully administered to prevent it from flowing back to the proximal artery. After the transarterial infusion, we embolized the artery with docetaxel-loaded HepaSphere. The endpoint of embolization was a significantly slower blood flow. The regimen used for each session as well as the adverse events induced by each regimen are described in Table 1. After the first session, CECT showed the diameter of the main tumour to be 49 mm (9.3% reduction). Grade 2 alopecia was observed after 3 weeks, which may be attributed to the docetaxel. We therefore used an adriamycin-based regimen instead of docetaxel in the second session. After the second session, the diameter of the main tumour decreased to 41 mm (16% reduction) and Grade 1 pigmentation appeared in an area of the left internal mammary artery, so the dose of adriamycin was decreased (from 20 to 10 mg) along with that of mitomycin C (from 4 to 2 mg). The treatment procedure was successfully performed in all the three sessions. The total doses of docetaxel-loaded HepaSphere were 9.75, 7.75 and 5.25 mg for the first, second and third sessions, respectively. No adverse events greater than Grade 3 and no signs of post-embolization syndrome were observed following the sessions.

**Figure 3.**
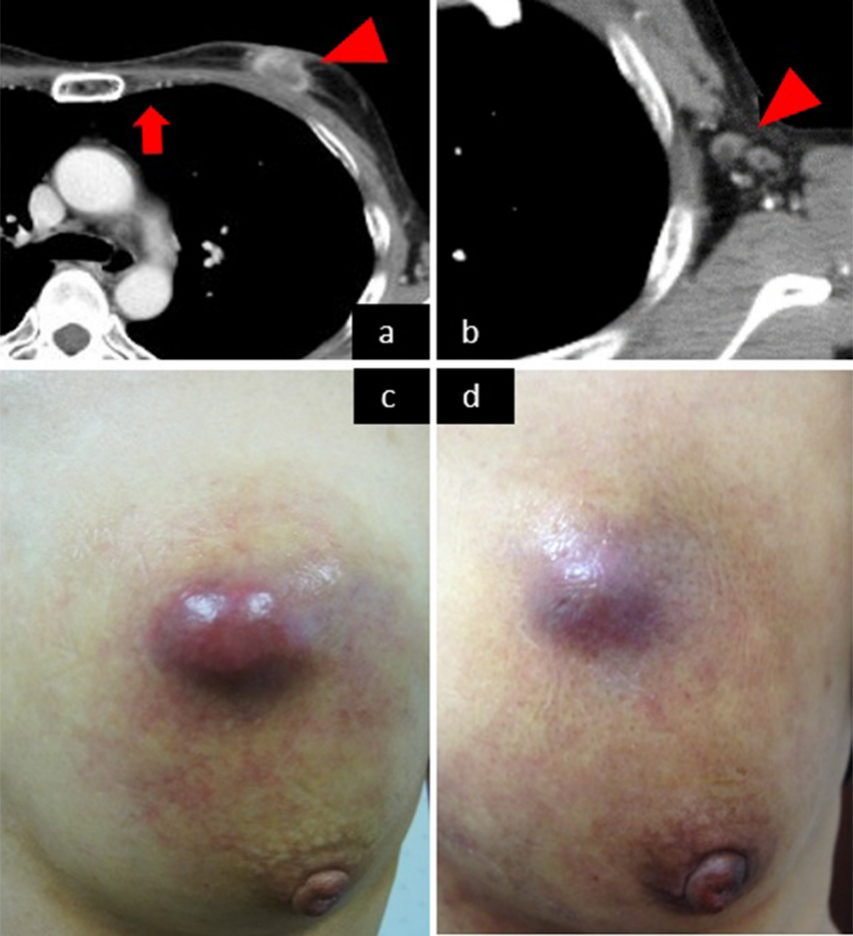
CT images and photographs demonstrating the effect of treatment. The primary lesion (arrowhead in a) and axillary lymph node (arrowhead in b) were reduced in size and the parasternal lymph node (arrow in a) disappeared after the third session. The skin lesion before treatment was reddish, oedematous and tense (c), which improved after the first treatment session (d).

CECT was performed after the third session of TACE. The primary lesion and axillary lymph node were 26 × 14 mm (37% reduction) and 10 mm, respectively (Figure 3a,b). CECT after three sessions showed that the parasternal lymph node had completely resolved (Figure 3a). The sharp pain and taut skin associated with the left breast tumour were also improved (Figure 3c,d).

**Figure 4.**
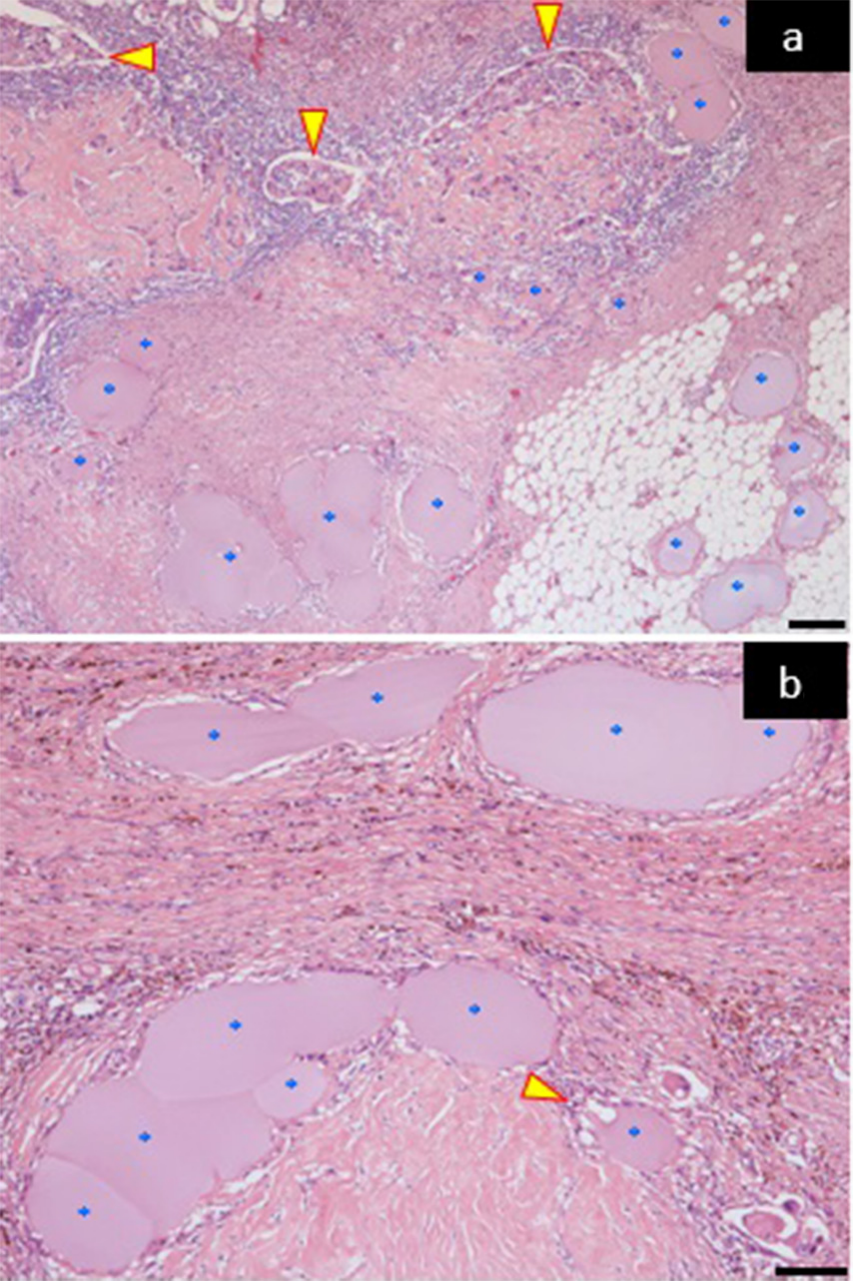
Photomicrographs of the surgically removed breast tissue. (a) The breast lesion tissue demonstrated residual and viable invasive ductal carcinoma of no special type (arrowheads) accompanied by fibrotic and inflammatory granulation tissue, along with a large number of arteries that were embolized with lavender-coloured spherical particles (asterisks). Haematoxylin and eosin staining; magnification: 4×; scale bar: 200 μm. (b) Several spherical particles with altered shapes appeared aggregated within the vessels and were closely attached to the inner walls of the vasculature (asterisks). One spherical particle appeared to have infiltrated from the vessels to the breast tissue, accompanied by infiltration of macrophages (arrowhead). Haematoxylin and eosin staining; magnification: 10×; scale bar indicates 100 μm.

The treatment effect motivated the patient to receive surgical intervention. In December 2014, left total mastectomy and axillary lymph node dissection were performed. Histopathologically, the resected breast lesion, which measured 2.5 × 1.3 cm, demonstrated residual and viable invasive carcinoma of no special type, infiltrating into the dermis but without invasion into the pectoral muscle. The daughter nodule had disappeared, and fibrotic and inflammatory granulation tissue was observed along with a large number of arteries that were embolized with spherical particles (Figure 4a). Meanwhile, the dissected axillary lymph node contained a large amount of multinucleated giant cells and macrophages, but no carcinoma cells were identified by both haematoxylin and eosin staining, and broad cytokeratin immunohistochemistry. The histopathological post-therapy effect was considered to be a mild response in the breast lesion (Grade 1a: mild changes in cancer cells regardless of the extent, and/or marked changes in less than one-third of cancer cells), and a complete response in the axillary lymph node lesion (Grade 3: necrosis and/or disappearance of all tumour cells, and/or the replacement of cancer cells by granulation, and/or fibrosis), according to the proposed criteria.^1^

The mean ± standard deviation of the minor axis of 17 randomly selected arteries embolized with spherical particles was 183.0 ± 96.5 μm (range: 71.3–451.5 μm). Several spherical particles with altered shape appeared aggregated within the vessels and were closely attached to the vascular inner walls. Furthermore, several spherical particles appeared to have infiltrated from the vessels to the breast tissue accompanied by infiltration of macrophages (Figure 4b). The patient has now been followed for 1 year after the surgery, with no signs of recurrence.

## Discussion

We succeeded in controlling LABC with TACE using spherical embolic material. To our knowledge, this is the first report regarding the activity of HepaSphere on the clinical outcome and histopathology of human breast cancer.

Systemic chemotherapy for primary, recurrent and metastatic breast cancer has been shown to be efficacious and is the standard therapy.^2^ However, in many cases, systemic chemotherapy is not indicated or is discontinued because of severe progressive disease, treatment failure, advanced age of the patient, multiple comorbidities, as well as patient refusal, as with our case. As a result, some patients leave their tumours untreated. In these cases, the tumours can enlarge, invade the chest wall, skin, pectoralis major muscle, and become ulcerated, subsequently resulting in metastasis to various organs and leading to death of the individual, or can even result in life-threatening haemorrhage.^3^ Breast cancers are hypervascularized tumours. When a massive haemorrhage occurs, it is difficult to control the bleeding. Our case suggests that TACE with spherical embolic material can be performed at an early stage to prevent these major complications.

Transarterial treatment can be traced back to the 1960s and has previously been used to provide tumours with high local drug concentrations, with fewer side effects than systemic chemotherapy. In 1975, Koyama et al^4^ reported decrease in the size of the tumour in all their 12 breast cancer patients following infusion from the internal mammary and subclavian arteries. In 1985, Morimoto et al^5^ reported a mean regression rate of 55% in 17 LABC patients. Their strategy was to use anticancer drug-loaded embolic agents so that the anticancer drugs remained in the tumour tissue, resulting in reduced side effects.^5^ The response rate of transcatheter arterial treatment for LABC was recently reported to be as high as 77.3–90.6%.^6–9^ In addition, Shimamoto et al^6^ demonstrated the efficacy and safety of the redistributed subclavian arterial infusion chemotherapy method, which uses a port system.

In breast cancer, feeding arteries are associated with each tumour site. In many cases, inner and outer breast tumours are mainly supplied by the internal mammary artery and the lateral thoracic artery, and the thoracoacromial artery, respectively. When there is difficulty in finding the feeding arteries (unlike in this case), a tourniquet has conventionally been used and is often found to be effective.^6^ The redistributed subclavian arterial infusion chemotherapy method also applies this principle.

A variety of regimens and doses of anticancer drugs have previously been reported for the treatment of breast cancer.^4,7–9^ In our opinion, similar to systemic chemotherapy, anthracycline and taxane are key drugs for breast cancer. Regarding the dose, a lower dose of these drugs is sufficient for transarterial chemoinfusion. If the tumour is neither significantly reduced in size nor in enhancement on CECT, which suggests necrosis, we recommend changing the regimen after one session to see if the carcinoma may be more sensitive to an alternative drug. If the tumour is unresponsive, increasing the dose of the chemotherapeutic drug should be avoided, because it is significantly more likely to increase adverse reactions.

TACE has been known as a minimally invasive therapy and is often preferred for its low rate of adverse events and complications compared with systemic chemotherapy. Pacetti et al^9^ reported on the safety of intra-arterial infusion for elderly patients with LABC.

Wang et al^7^ have described the rate of complications of treatment with intra-arterial infusion chemotherapy for LABC. In a total of 53 patients, severe complications included ipsilateral upper extremity atrophy in two cases and necrosis of local skin in one case. Controlling the pressure of the tourniquet and velocity of drug administration appeared to be crucial in avoiding such complications.

Spherical embolic materials have been used for hepatocellular carcinoma, malignant tumour haemorrhage, uterine myoma and arteriovenous malformation. HepaSphere is a new type of spherical embolic material that consists of superabsorbent polymer microspheres and has three well-known unique characteristics. First, they are deformable. The shape of the microspheres after embolization was not perfectly spherical but oval (Figure 4). Second, they are expandable. The microspheres absorb fluid and swell, and the final size depends on the osmolality of the liquid in which they are diluted. de Luis et al^10^ performed a study embolizing swine kidney arteries using HepaSphere. Before embolization, they diluted particles with pure contrast material. The final size of the particles was 230 ± 62.5 µm for particles measuring 50–100 µm in the dry state. On the other hand, in our case, in spite of using the same size dry-state HepaSphere particles, the final size of the particles was 183 ± 96.5 µm, which was a smaller size than seen in the previous study because we diluted them using the swelling suppression method, that is, the 1 to 4 method (a mixture of 10% NaCl and contrast material at a ratio of 1 : 4). We strongly recommend this method because smaller microspheres will be more selective and be able to reach more distal sites. Third, they are loadable. Particles are injected selectively into the feeding arteries and release the anticancer drug gradually. To date, doxorubicin, irinotecan and docetaxel have been loaded in the microspheres. Docetaxel has been shown to be one of the most effective drugs for breast cancer. However, severe adverse events, including alopecia or neutropenia, have occurred in cases of systemic chemotherapy, which reduce the patients’ quality of life. On the other hand, a low dose of docetaxel is sufficient for loading HepaSphereparticles to obtain an effect after performing TACE. Few studies, however, have been published about docetaxel-loaded HepaSphere. Additional *in vivo* and *in vitro* experiments are required to clarify the behaviour of docetaxel-loaded HepaSphere.

## Conclusions

We succeeded in reducing the size of LABC by TACE with spherical embolic material. The present case suggested that TACE with HepaSphere may be the treatment of choice for patients who refuse conventional treatment or are at high risk of adverse events upon receiving conventional treatment, from both clinical and pathological viewpoints.

## Learning point

1. Selective chemoembolization with HepaSphere for extrahepatic tumours results in a reduction in tumour size, and is safe with minimal adverse events.

## Consent

Written informed consent was obtained from the patient for publication of this case report, including the accompanying images.
